# Grain boundaries and coincidence site lattices in the corneal nanonipple structure of the Mourning Cloak butterfly

**DOI:** 10.3762/bjnano.4.32

**Published:** 2013-05-02

**Authors:** Ken C Lee, Uwe Erb

**Affiliations:** 1 Department of Materials Science and Engineering, University of Toronto, Toronto, Ontario, M5S 3E4, Canada

**Keywords:** butterfly-eye structure, coordination defects, sigma grain boundaries

## Abstract

In this study the highly ordered corneal nanonipple structure observed on the Mourning Cloak butterfly (*Nymphalis antiopa)* is analyzed with a particular emphasis on the high-angle grain-boundary-like defects that are observed between individual nanonipple crystals. It is shown that these grain boundaries are generated by rows of topological coordination defects, which create very specific misorientations between adjacent crystals. These specific orientations form coincidence site lattices, which (i) have unit cells larger than the unit cell in each individual crystal and (ii) extend from one crystal to the next, effectively creating order over areas larger than the individual crystals. A comparison to similar coincidence site lattices in engineering materials is made and the importance of such arrangements in terms of nipple packing density, corneal lens curvature and potential optical properties is discussed.

## Introduction

The structure of moth and butterfly eyes consist of many repeating unit cells referred to as ommatidia. Thousands of ommatidia are closely packed together and typically form a nearly spherical or semispherical compound eye with the well-known surface facet structure [1]. Each ommatidium is composed of a corneal lens, a crystalline cone, a rhabdom and retinula cells. The signals detected by thousands of ommatidia are processed neurologically in the brain to form a complete image. The corneal lens incorporates chitin, a long chained semicrystalline natural polysaccharide with a refractive index of about 1.52. It is well known that the outer surfaces of the corneal lens of some moths and butterflies are covered with so-called corneal nipples, nanometer-sized protuberances distributed over the surface in patterns with varying degrees of regularity [2–6]. In an earlier study these nipple arrays were classified in different categories depending on nipple height, which can vary from less than 50 nm to over 200 nm [3]. The advantages of compound eyes with corneal nipple structures, compared to flat lens surfaces observed in many other insects, have been discussed in numerous studies, e.g., [5–7]. Most importantly, these structures reduce the reflection of light from the surface of the eye, due to the gradient in the refractive index in the near surface region. This is important for nocturnal insects such as moths, because it gives a better low-light vision (moth-eye effect). The reduced light reflection also provides an antiglare effect, which provides some protection from predators. In recent years many artificial moth-eye-type surfaces have been developed as antireflection, antiglare surfaces by various methods such as photoresist patterning, porous aluminium oxide templating or direct reproduction, e.g., [7–9]. With respect to the topological arrangements of the nanometer sized nipples, both completely irregular and highly ordered structures have been observed on different species [2,6]. In an ongoing study, we analyze the highly ordered nipple structure of the Mourning Cloak butterfly (*Nymphalis antiopa*) in more detail and from a crystallography/defect-structure point of view. The results of this investigation will be published shortly [10]. Briefly, this study has shown that the highly ordered hexagonal structure is made up of nipples with average diameters on the order of 150 nm and a unit cell lattice parameter of about 200 nm. Perfect crystals with sizes on the order of a few micrometers cover the entire surface of each ommatidium. The crystals are separated by grain-boundary type defects created by rows of 5–7 coordination defects. While an individual 5–7 coordination defect in a perfect crystal creates a dislocation-type defect, rows of these defects with relatively large spacing form low-angle grain boundaries. With decreasing spacing between 5–7 coordination defects, high-angle grain boundaries are formed for which the misorientation between adjacent crystals is more than 10 degrees.

In the current investigation the type and nature of these high-angle grain boundaries was studied in more detail. It will be shown that these boundaries have preferential misorientation angles, such that adjacent crystallites are arranged in specific coincidence site lattices (CSLs). A comparison to similar CSLs observed in engineering materials will be made and the purpose and potential advantages of such unusual nipple array structures will be discussed in the current report.

## Results and Discussion

### Eye structure

The Mourning Cloak butterfly (*Nymphalis antiopa*) is a common butterfly in North America. It has a wing span of 4–8 cm and shows beige-yellow edges and several blue spots on brown colored wings.

**Figure 1:**
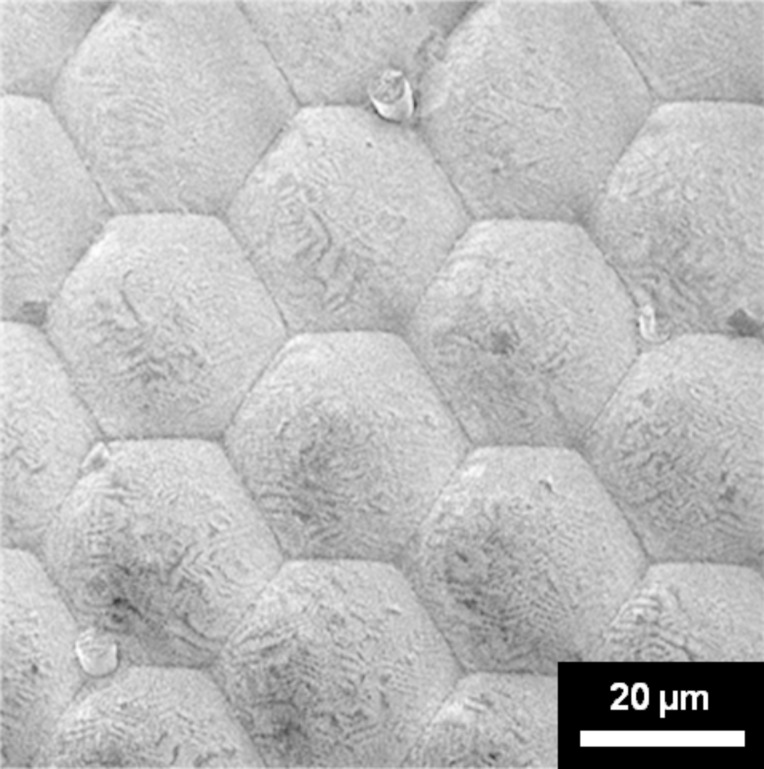
Scanning electron micrograph showing the facet structure of the Mourning Cloak butterfly. Note the bases of fractured hair at some of the triple junctions (magnification: 800×).

A low magnification scanning electron micrograph of one of the eyes is shown in Figure 1. The hexagonal shape of the ommatidia is clearly visible with each facet measuring about 25 µm across. The entire eye of the Mourning Cloak butterfly is covered with randomly distributed tiny hairs (about 4 µm in diameter), which usually grow at the triple junctions between the ommatidia. In Figure 1, the bases of fractured hairs are visible on three of the triple junctions. Figure 1 also shows areas with contaminations/local damage, which are likely the result of the collection and the handling and/or preservation processes of the specimen.

**Figure 2:**
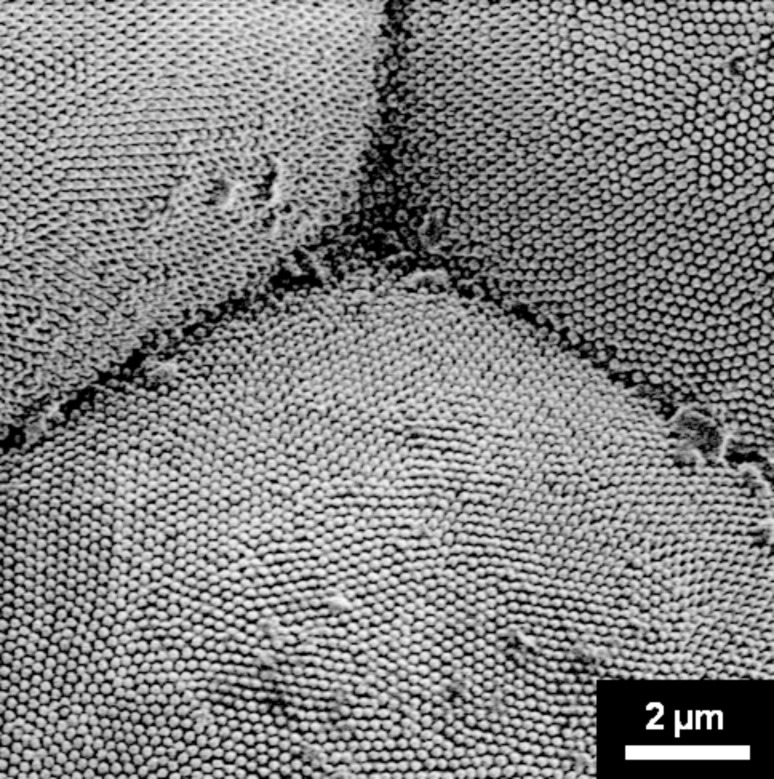
Triple junction in the facet structure and nanonipples in three adjacent facets (magnification: 6,000×).

Figure 2 shows a triple junction between three ommatidia at higher magnification. In this micrograph the nanonipple structure on each of the facets is clearly visible. At the triple junction and the three facet boundaries the nipple structure is highly irregular showing no long-range order in their arrangement. However, with increasing distance from the triple junction and the facet boundaries, considerable structural order in the nipple arrangements can be seen as crystals, i.e., areas with close-packed, hexagonal nipple arrangements. These crystals can be several micrometers in diameter and are separated by topologically disordered or defective regions across which the orientation of the ordered regions changes by various rotational angles. In an earlier study on nipple arrangements on various butterflies similar structures were referred to as local domain arrangements [6].

**Figure 3:**
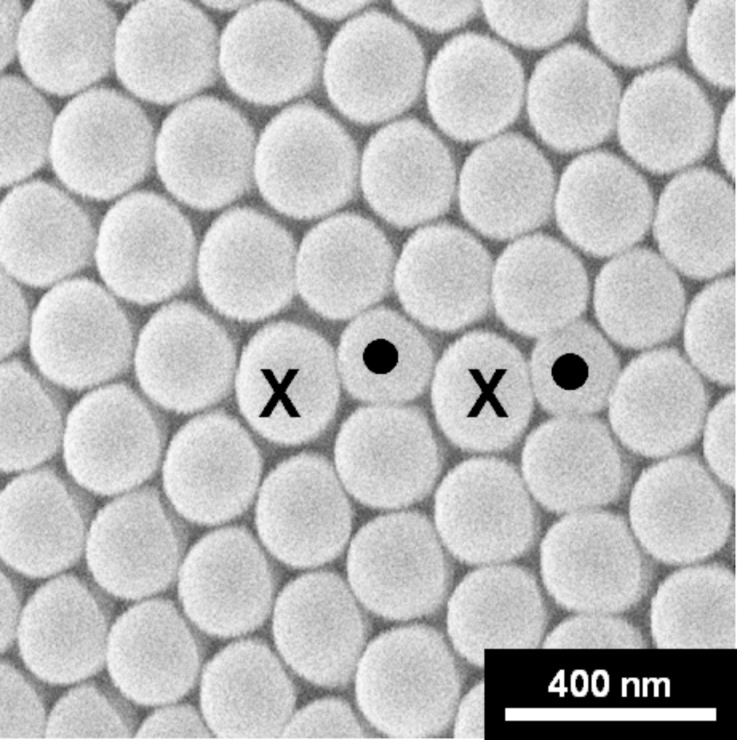
Topological disorder in the hexagonal nipple structure showing two pairs of 5–7 (“**•**”–“**X**”) coordination defects (magnification: 52,000×).

For the remainder of this study, nipple arrangements located at least 4 µm away from triple junctions and facet boundaries in contamination-free areas of the eye were selected. Figure 3 shows a high magnification micrograph of the nipple arrangement in one of the highly ordered regions. While most of the nipples are arranged in a regular hexagonal close-packed pattern, the structure is defective at the points marked with “•” and “**X**”. We refer to these defects as 5–7 coordination defects, because the normal coordination number of six for the defect-free regions of the nipple arrangements is locally changed to five or seven nearest neighbors. It was observed that the nipples with seven nearest neighbors were usually slightly larger than the average nipple diameter (150 nm) while the nipples with five nearest neighbors were slightly smaller. These coordination defects usually occur in pairs. Only a small number of isolated 5-fold coordination defects were found. One pair of 5–7 coordination defects creates a dislocation-type defect in the crystalline structure [10].

Moreover, many 5–7 coordination defects were arranged in rows creating defects that can be described as high-angle grain boundaries [10]. In the current study we analyzed the type of these high-angle boundaries in more detail.

**Figure 4:**
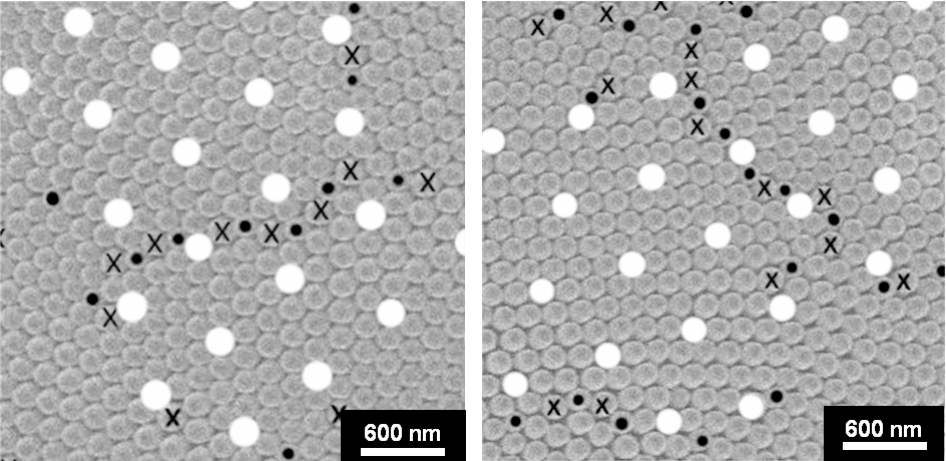
Examples of Σ13 grain boundaries between crystals (domains) in the nipple structure consisting of rows of 5–7 coordination-defects. White circles indicate nipple positions belonging to a coincidence site lattice (CSL) superlattice (magnification: 20,000×).

Figure 4 shows two different regions with rows of 5–7 coordination defects again marked with “•” and “**X**”. It can be seen that these defect rows are clearly grain-boundary-type defects across which the orientations of otherwise defect-free crystals change. What is quite remarkable in both cases shown in Figure 4 is that some sort of superlattice can be identified as indicated by the nipples marked in white. This superlattice has the property of a regular repeat pattern with a unit cell several times larger than the unit cell in each of the crystals. The superlattice extends over adjacent crystals and is not interrupted by the grain boundaries separating two crystals. Another feature of the superlattice is that it subdivides the nipple array into regions of relatively good superlattice fit that are much larger than the crystal/domain size in the nipple array. It can be noted in both examples shown in Figure 4 that the superlattice is not 100% perfect for all nipple positions. Instead, some minor deviations from the perfect superlattice position are found for some of the nipples. The significance of these deviations will be discussed later.

### The coincidence site lattice (CSL)

Superlattice structures such as those shown in Figure 4 have been studied in crystalline materials for several decades in the context of grain boundary analysis. For example, in 2D crystal structures produced by bubble rafts [11] or 3-D polycrystalline materials [12–13] such superlattices were studied for many different grain boundaries. Kronberg and Wilson [12] were likely the first researchers to point out the significance of superlattices for crystals rotated about the common <111> crystallographic direction and the preferred misorientations of crystals separated by <111>-type grain boundaries in recrystallized copper. In this work the term Σ was introduced to characterize such preferred orientations and the importance of Σ for grain boundaries in copper was discussed. The Σ-value is the size ratio of the superlattice unit cell divided by the original lattice unit cell. It should be noted that the motif arrangement of the closest-packed (111) plane in face-centered cubic copper is identical to the hexagonal close packed plane in the 2-D crystal structure of the butterfly nipple structure arrangement.The work by Kronberg and Wilson was the foundation for numerous studies over the past six decades, which looked at the significance of so-called coincidence site lattices (CSLs) for grain-boundary structure and property analyses in engineering materials.

Ranganathan [13] derived the following mathematical relationships for the generation of general CSLs about any common rotation axis <*hkl*>, not only the <111> direction.

[1]
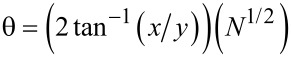


[2]
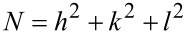


[3]
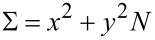


where θ is the misorientation angle between the two adjacent crystals, *h*, *k*, *l* are the Miller indices of the common rotation axis, and *x*, *y* are the coordinates of superlattice sites.

These CSL descriptions can be applied to both 2-D and 3-D crystal arrangements. It should be noted that all grain boundaries can be described by a Σ value. However, with increasing Σ number the physical significance of the superlattice becomes questionable as the unit cell of the lattice becomes very large. Many studies have shown that grain boundaries in materials associated with relatively low Σ values (small CSL unit cells) show special properties such as low energy or reduced susceptibility to intergranular degradation (e.g., intergranular corrosion, cracking, segregation) as summarized in several books, e.g., [14–15]. It was further shown that the special properties of grain boundaries were even maintained for small angular deviations (Δθ) from the special misorientations (θ). Several criteria were introduced for permissible angular deviations up to which the special properties of grain boundaries are maintained [16–19]. The most commonly used criterion is the Brandon criterion [16], which states

[4]
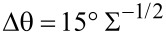


Table 1 summarizes all possible superlattices with Σ ≤ 19 that can be generated for increasing misorientation values up to θ = 60° about the <111> crystallographic axis, together with the corresponding deviation angles Δθ according to the Brandon criterion.

**Table 1:** Σ-values for CSL lattices generated by misorientations θ about the common <111> rotation axis together with Δθ values according to Equation 4.

Σ	θ (°)	Δθ (°)

**19**	13.2	3.4
**7**	21.8	5.7
**13**	27.8	4.2
**13**	32.2	4.2
**7**	38.2	5.7
**19**	46.8	3.4
**3**	60.0	8.7

For a polycrystalline aggregate with crystals in random crystallographic orientations it has been shown that the fraction of grain boundaries with low Σ values is on the order of 10% [20]. In other words, a random material contains about 10% of grain boundaries with potentially enhanced resistance to intergranular degradation. In 1984, Watanabe suggested that the overall resistance of a material to intergranular degradation could be increased enormously by “grain-boundary design and control” which involves materials processing routes that increase the number of special boundaries [21]. This led to the new field of grain-boundary engineering, which has been successfully applied for many metals and alloys [22–24].

### CSL structures on Mourning Cloak butterfly eyes

**Figure 5:**
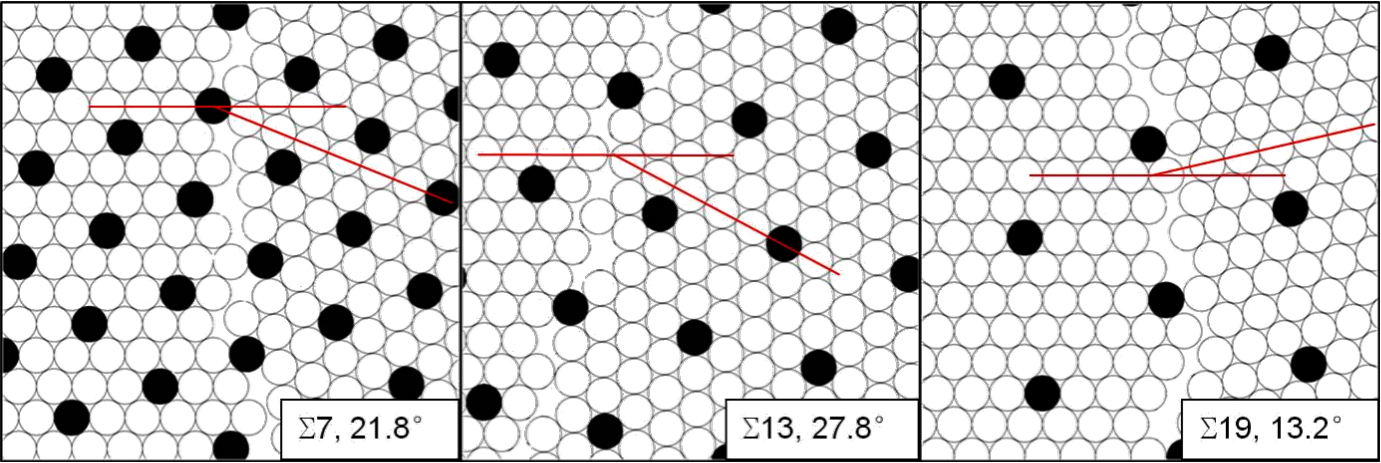
Coincidence site lattices for Σ = 7, Σ = 13 and Σ = 19.

Returning to the importance of CSL grain boundaries for the 2-D nipple crystal structures found on the Mourning Cloak butterfly eye, Figure 5 shows three different special <111> tilt grain boundaries for three crystal rotations about the common <111> rotation axis. All three rotations correspond to low Σ values of Σ = 7 for θ = 21.8°, Σ = 13 for θ = 27.8° and Σ = 19 for θ = 13.2° (Table 1). It should be noted that in these figures the open circles show motif positions in the two crystals while the filled circles are the positions of CSL motifs belonging to both crystals. It can be clearly seen that the CSLs in all three cases extend across the grain boundaries over both crystals. The figures also show that the unit cell of the CSLs becomes larger with increasing Σ value. It should be pointed out that in Figure 5 the size of all motifs is the same, while in the nanonipple arrangement of the Mourning Cloak butterfly the grain boundary is introduced by rows of 5–7 coordination defects in which the nipple sizes vary to some extent. However, for the crystallographic description of the superlattice the actual nipple size distribution along the grain boundary is of secondary importance.

**Figure 6:**
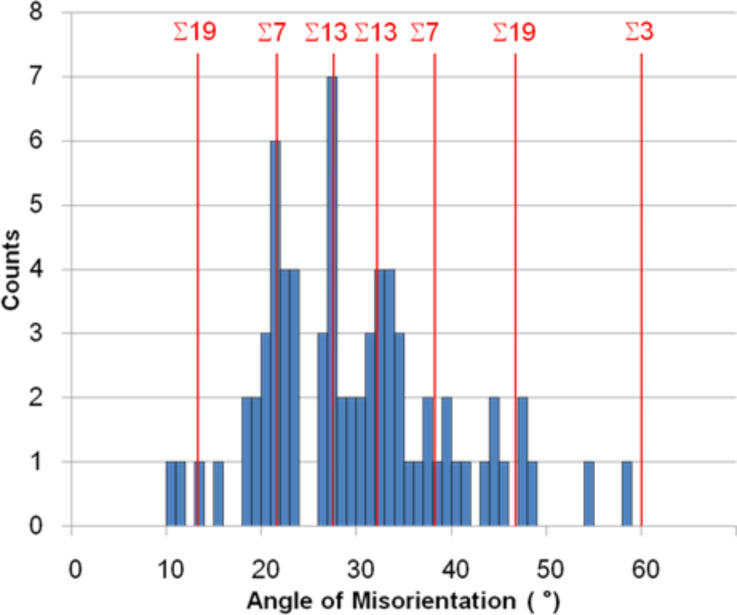
Histogram showing the misorientations around the common <111> direction between adjacent crystals in the Mourning Cloak nanonipple structure.

In order to assess the statistical significance of CSL boundaries within the nipple arrangements of the Mourning Cloak butterfly a total of 73 grain boundaries such as the ones shown in Figure 4 were analyzed. The results of this analysis are presented in Figure 6, which shows the number of boundaries found as a function of their misorientation θ about the common <111> tilt axis. Also shown in this Figure are the positions of various low Σ grain boundaries. This figure clearly demonstrates a strong preference for Σ7 and Σ13 boundaries, and even perhaps Σ19. Surprisingly, there is no preference for Σ3 boundaries expected at an angle of misorientation of 60°. Many engineering materials show a high preference for Σ3 boundaries [14–15]. Note that when the Brandon criterion is applied, all boundaries fall within the angular deviation given in Equation 4. In other words, all 73 boundaries can be considered special boundaries.

**Figure 7:**
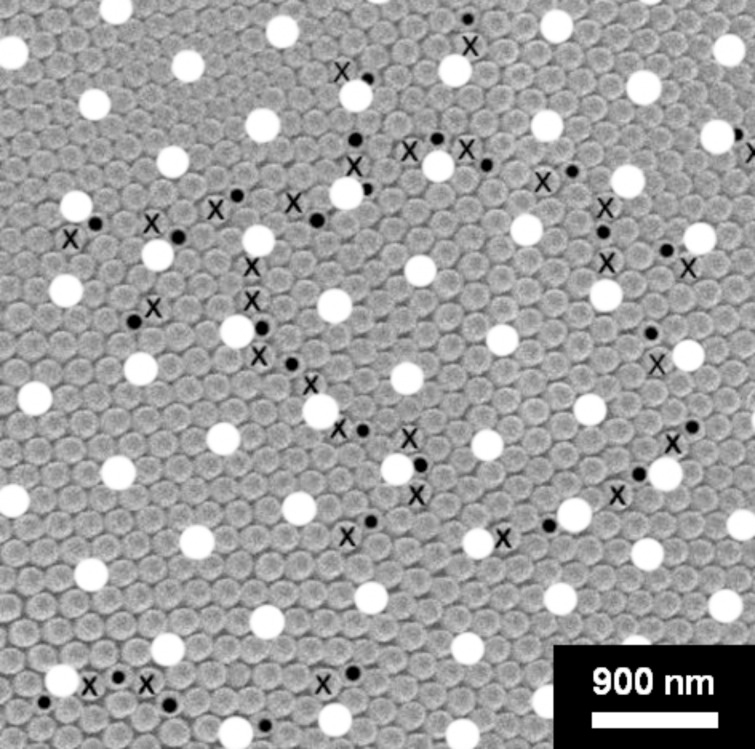
Extended Σ13 coincidence site lattice covering several crystals (magnification: 13,000×).

It was observed that the nipple arrangement was not perfect for all CSL positions shown in Figure 4 and that some deviations did occur. This becomes more obvious when the area of analysis is increased as shown in Figure 7.

**Figure 8:**
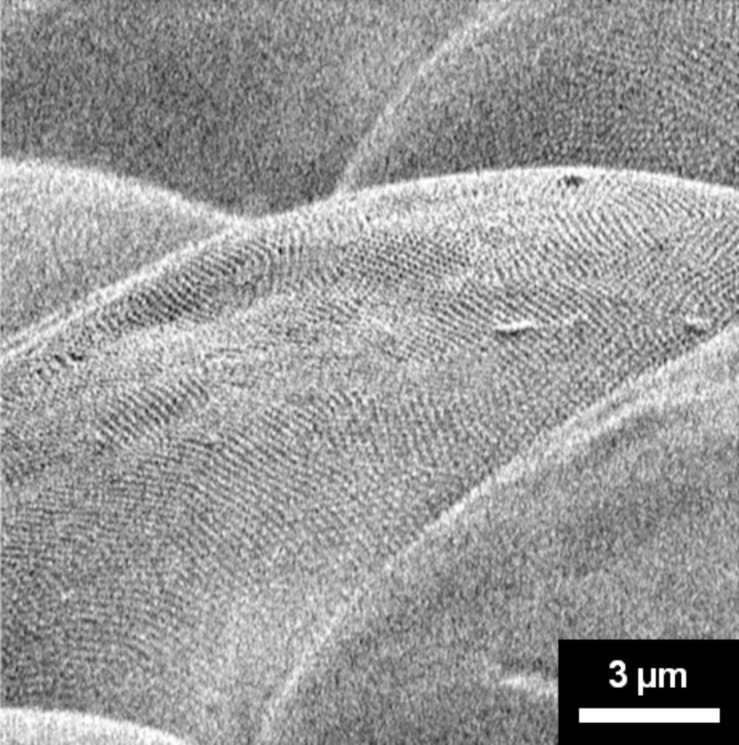
Several facets at very high tilt angle showing the curvature on each facet surface (magnification: 4,000×).

This figure clearly shows considerable distortions in the superlattice unit cell and a fairly pronounced waviness in rows of closest packed CSL lattice lines. However, this is perhaps not surprising because the SEM micrographs are 2-D projections of the ommatidia surfaces, which actually show a considerable curvature. This is observed in Figure 8, which is a micrograph of several ommatidia taken at a relatively high tilt angle to visualize their surface curvature. The radius of curvature can be estimated to be on the order of 16–20 µm. In addition, many ommatidia show some localized depressions, likely due to dehydration. It is therefore expected to see distortions of the superlattices shown in Figure 4 and Figure 7 as these are projections of curved 2-D crystals. In other words, the superlattice fit may actually extend over much larger areas if this curvature is taken into consideration.

In summary, the 2 mm hemispherical eyes of the Mourning Cloak butterfly consist of several thousand ommatidia, each covered with thousands of nanonipples. A total of about 100 million of these nipples are arranged in closest packed hexagonal crystals separated by grain boundaries. A small sample of 73 of these boundaries has shown that they are mainly of the Σ7 and Σ13 type and perhaps also Σ19 boundaries. These boundaries consist of rows of 5–7 coordination defects, which are generated by using slightly larger/smaller nipple sizes than the average nipple size. These Σ-related superlattices extend over areas larger than the average crystal size.

When looking at these surprising results two questions arise: (i) what is the purpose of this peculiar nipple arrangement, and (ii) what benefit does the Mourning Cloak butterfly derive from these structures? Perhaps it is instructive to first look at nipple arrangements on the moth eye, which has been studied much more extensively in the past. The moth eye has very similar nipple arrangements to the Mourning Cloak butterfly, although a detailed analysis of the exact arrangements of nipples along crystal interfaces has not been presented yet. One of the earliest theories [2] for the purpose of the nanonipples on the moth eye is that they effectively decrease the reflection of light from the eye because of the gradient in the refractive index as light travels from air (*n* = 1.0) to the ommatidia (chitin, *n* = 1.52). Thus, more light enters the eye, which is beneficial for moths as they are nocturnal and active in low light conditions. However, the Mourning Cloak is a diurnal butterfly, active during the day where low-light vision improvement may not be the most important. Nevertheless, the Mourning Cloak butterfly could benefit from less glare of the eye due to reduced reflection, which perhaps provides some protection from predators. However, a reduced eye reflection does not necessarily require ordered nipple arrangements.

Space filling due to the curved surfaces of the ommatidia could be another reason for the preference of coordination defects. If the intent is to create the highest possible density of nipples on the eye, a perfectly flat closed-packed crystal must include some sort of defects when curvature is introduced. In fact, nature uses this trick quite often to introduce curvature into hexagonal closely packed structures. For example, the combs of honey bees and social wasps, which also make use of space-filling hexagonal patterns, show interruptions to adjust for curvature, confined boundary conditions, or transition zones from smaller to larger cells [1]. Sometimes four-sided cells are found, but most commonly it is a row of five-sided cells that accommodates such obstructions. However, configurations of rows of several 5-fold coordination defects were not found in the nipple structures of the Mourning Cloak butterfly.

If the purpose of the CSL lattices is to create special properties of the eye, it is likely associated with an optical function. One such function could be a diffraction capability making the eye sensitive to diffraction phenomena for certain wavelengths. Table 2 shows the distances between rows of CSL sites for the three Σ boundaries Σ7, Σ13 and Σ19.

**Table 2:** Distances between coincident sites on the eye of the Mourning Cloak butterfly.

Σ	grating distance (nm)

**7**	*d*_1_ = 529
	*d*_2_ = 917

**13**	*d*_1_ = 721
	*d*_2_ = 1249

**19**	*d*_1_ = 872
	*d*_2_ = 1509

If diffraction of light is indeed a primary role of the superlattices formed on this butterfly eye, the various CSLs could provide diffraction gratings that are sensitive to selective wavelengths in the visible and infrared wavelength range. It is suggested that a future study should be concerned with optical measurements to explore if there is indeed any wavelength sensitivity due to diffraction as a result of this unusual structure.

Although completely different in nature, the 5–7 coordination defects found as the most common defect in the Mourning Cloak butterfly eye are topologically similar to some of the defects found in other structures with closest packing. For example, in hexagonal graphene and carbon nanotubes, Stone–Wales 5–7 defects [25–27] have been found in numerous studies. Rows of 5–7–7–5 defects and other combinations have also been associated with grain boundaries in such structures [27]. Coordination defects are also common in other curved surfaces with closest packed structures such as polymer colloids assembled on water or oil droplets [28–29]. The importance of 5–7 topological coordination-number defects has further been discussed for the cases of grain growth in polycrystalline materials and the stability of periodic cellular structures [30–31]. All these examples show that, from a topological point of view there are similarities between inorganic materials and living structure patterns that should be further explored, as suggested earlier [32].

## Experimental

The Mourning Cloak butterfly used in the current study was received from Thorne’s Insect Shoppe Ltd, London, Ontario. It was collected in July 2008 at Miner’s Bay, Ontario. The head of the butterfly was separated, mounted on a holder and carbon coated. Scanning electron micrographs were taken in a Hitachi 4500 field emission scanning electron microscope operated at 5 kV acceleration voltage. Micrographs were taken at various locations in contamination/macro-defect-free regions.
